# „Digitalisierung mit der Brechstange“? Auswirkungen der Pandemie auf Digitalisierungsprozesse und digitale Ungleichheiten an österreichischen Schulen

**DOI:** 10.1007/s35834-022-00347-5

**Published:** 2022-07-21

**Authors:** Gerlinde Janschitz, Elisabeth Zehetner, Karina Fernandez

**Affiliations:** 1 Deutsches Jugendinstitut, Nockherstraße 2, 81541 München, Deutschland; 2 Institut für Educational Governance, Pädagogische Hochschule Steiermark, Ortweinplatz 1, 8010 Graz, Österreich

**Keywords:** Covid-19, Digitale Ungleichheit, Digital Divide, Distance Learning, Covid-19, Digital Inequalities, Digital Divide, Distance Learning

## Abstract

Die Covid-19 Pandemie traf im Frühjahr 2020 auf die österreichische Schullandschaft und stellte Österreichs Schulen vor eine Ausnahmesituation. Die darauffolgenden Phasen des Distance Learning trieben auch Digitalisierungsprozesse im schulischen Bereich voran. In diesem Kontext kommt sowohl der technischen Ausstattung als auch den digitalen Kompetenzen der Schüler*innen große Relevanz zu – und dies birgt die Gefahr, dass sich durch einen Digital Divide bereits bestehende Ungleichheiten im Bildungssektor zuspitzen. Der vorliegende Beitrag beleuchtet daher mittels Daten aus einer Interviewstudie mit Lehrkräften, Schulleiter*innen, Mitarbeiter*innen psychosozialer Unterstützungssysteme und Schüler*innen Digitalisierungsprozesse im schulischen Bereich während der Pandemie. Diese Prozesse werden vor dem Hintergrund der Diskussion um digitale Ungleichheiten im Schulkontext diskutiert. Die Ergebnisse der vorliegenden Studie zeigen, dass es durch den Covid-bedingten Fernunterricht zu einem deutlichen Ausbau und einer gewissen Konsolidierung digitaler Formen des Lehrens und Lernens kam. Dennoch bleiben Probleme bestehen, die mit strukturellen Ungleichheiten verbunden sind. Zu Aufholprozessen für sozial benachteiligte Schüler*innen kam es nicht. Vielmehr schreiben sich jene Faktoren, die bereits vor der Pandemie für die tiefgreifenden Ungleichheiten im österreichischen Bildungssystem ausschlaggebend waren – wie sozioökonomischer Status, Bildungsstand der Eltern und kulturelles Kapital – auch im Distance Learning und im Arbeiten mit digitalen Medien weiter fort.

## Abstract (en)

The Covid 19 pandemic hit the Austrian school system in spring 2020, bringing a range of new challenges. The following phases of school closures and distance learning also promoted digitalisation processes in schools. In this context, technical equipment and digital competences of students gain importance, which bears the risk that a digital divide deepens already existing inequalities in education. This article therefore examines digitalisation in schools during the pandemic and takes a closer look at digital inequalities during distance learning. We use data from an interview study with teachers, school administrators, employees of psychosocial support systems at schools, as well as students. The findings of this study show that Covid-related school closures and distance learning go along with an expansion and a certain consolidation of teaching and learning by digital means. Yet, we see persisting problems which are linked with structural inequalities. There were no catch-up processes for socially disadvantaged students as a result of digitalisation. Rather, the factors that were already decisive for the profound inequalities in the Austrian education system before the pandemic, such as socioeconomic status, educational level of the parents, and cultural capital, continue to be of major importance for distance learning and the use of digital media in schools.

## Einleitung

Die Covid-19 Pandemie traf im Frühjahr 2020 auf die österreichische Schullandschaft und stellte Österreichs Schulen vor eine Ausnahmesituation. Obwohl bis dahin schon länger ein breiter öffentlicher Diskurs über die Digitalisierung im Schulbereich geführt wurde, erfolgte nunmehr innerhalb von kurzer Zeit eine umfassende Umstellung auf Distance Learning^1^, bei dem digitale Medien für eine Mehrzahl der Schulen eine zentrale Rolle spielten – eine Situation, die diese meist völlig unvorbereitet traf. Im weiteren Verlauf der Pandemie erfolgte zwar nicht zuletzt im Bereich digitaler Lehre eine gewisse Normalisierung, dennoch bleiben große Herausforderungen bestehen, insbesondere was Bildungsungleichheiten betrifft. So deuten aktuelle Befunde darauf hin, dass bereits bestehende soziale Ungleichheiten im Bildungssystem durch die Covid-19 Krise verstärkt werden (Anger und Plünnecke 2021; Helm et al. 2021b; Maaz und Eickelmann 2021; Steiner et al. 2021). Entscheidend in dem Zusammenhang ist nicht zuletzt, dass die soziale Selektivität des Bildungsertrages in dem Ausmaß steigt, in dem dieser von der familiären Unterstützung abhängig ist (Maaz et al. 2011). Diese Befunde gewinnen vor dem Hintergrund der Covid-19 Pandemie an Brisanz – ganz besonders für die Phasen des Distance Learning: Neben Wissen und (digitalen) Kompetenzen der Eltern spielen auch die technische Ausstattung und räumliche Rahmenbedingungen eine Rolle.

Dass Fragen der Digitalisierung bei Bildungsungleichheiten eine wichtige Rolle spielen, ist jedoch kein neues Phänomen, das erst durch die Pandemie entstanden ist. Vielmehr wird im wissenschaftlichen wie auch im öffentlichen Diskurs schon seit den späten 1990er-Jahren und insbesondere mit Beginn des neuen Jahrtausends unter dem Schlagwort „digital divide“ (Norris 2001; DiMaggio et al. 2004) darauf Bezug genommen, dass der Zugang zu und die Nutzung von neuen Technologien von unterschiedlichen soziodemografischen und ökonomischen Faktoren abhängig sind. Diese Faktoren können zur Entstehung digitaler Ungleichheiten führen, die auf bereits bestehenden sozialen Ungleichheiten aufbauen (Zillien 2009).

Der vorliegende Beitrag verfolgt das Ziel, Digitalisierungsprozesse im schulischen Bereich während der Pandemie näher zu beleuchten und diese im Rahmen der Diskussion um digitale Ungleichheiten im Schulkontext aufzugreifen. Dabei stehen die folgenden Fragestellungen im Fokus:

- Welche Entwicklungen und Dynamiken zeigen sich im Verlauf der Covid-bedingten Umstellung auf digitalen Unterricht und Distance Learning an österreichischen Schulen? Was sind die Herausforderungen sowohl für Lehrer*innen als auch für Schüler*innen?
- Auf welchen Ebenen zeigt sich digitale Ungleichheit im schulischen Kontext während der Covid-19-Krise? Welche Aspekte (z. B. Ausstattung, Kompetenzen) sind besonders relevant und wie sind sie mit allgemeinen sozialen Ungleichheiten verflochten?

Bisherige Forschungsbefunde zum Thema beziehen sich vor allem auf quantitatives Datenmaterial. Der vorliegende Beitrag widmet sich diesen Fragestellungen auf Grundlage einer explorativen qualitativen Studie, um diese Zusammenhänge im Detail zu illustrieren und vertiefend zu beleuchten.

Nach Darstellung der aktuellen Forschungslage folgt im Ergebnisteil die Beschreibung zentraler Befunde einer Interviewstudie, die im Zeitraum von Herbst 2020 bis zum Sommer 2021 durchgeführt wurde. Dabei werden in einem ersten Schritt Strategien der Covid-bedingten Umstellung auf digitalen Unterricht sowie die Ausgestaltung und Folgen des Distance Learning analysiert. In einem zweiten Schritt folgt die Beschreibung unterschiedlicher Facetten digitaler Ungleichheiten im schulischen Kontext, deren Bedeutung vor dem Hintergrund der anhaltenden Pandemie in einem abschließenden Fazit kritisch diskutiert wird.

## Stand der Forschung: Digitalisierungsprozesse und Ungleichheiten im Schulbereich während der Pandemie

Die Covid-bedingten Schulschließungen gingen mit einer Umstellung des schulischen Präsenzbetriebes auf unterschiedliche Konzepte des Distance Learning einher, die sich seit nun mehr fast zwei Jahren der Pandemie ausdifferenziert und zum Teil konsolidiert haben. So wurden 87 % der deutschen Schüler*innen aufgrund der Corona-bedingten Schulschließungen 2021 digital unterrichtet bzw. wurden ihnen digitale Lehrangebote zur Verfügung gestellt (Initiative D21 2021, S. 2). Die Übermittlung der Lehrinhalte fand zum Großteil über E‑Mail sowie Videokonferenzen statt, wobei insbesondere die Abhaltung von Videokonferenzen über Programme wie Teams im Vergleich zu 2020 stark zugenommen hat (von 44 auf 63 %) (Initiative D21 2021, S. 6). Für Österreich zeigt eine Studie von Steiner et al. (2021, S. 33ff.) ebenfalls einen Anstieg der Nutzung von Videokonferenzen im schulischen Kontext über den Verlauf der Pandemie hinweg. So nutzten 55 % der befragten Lehrpersonen während der ersten Schulschließungsphase im Mai 2020 Videokonferenzen im Distance Learning, die damit weitaus seltener zum Einsatz kamen als Schulbücher (87 %), Videos (78 %) und Arbeitsblätter auf Papier (65 %). In der zweiten (partiellen) Schulschließungsphase Ende 2020 verwendeten bereits 89 % der Lehrkräfte Videokonferenzen (Steiner et al. 2021, S. 33). Bei der Nutzung digitaler Medien im Distance Learning gilt es jedoch zwischen den unterschiedlichen Schulstufen zu differenzieren. So wurden in Volksschulen Aufgaben vorwiegend in Papierform bereitgestellt, während vor allem ältere Schüler*innen synchronen Online-Unterricht erhielten (Postlbauer und Helm 2021).

Herausfordernd für die Schüler*innen im Distance Learning sind zahlreichen Studien zufolge vor allem das Fehlen einer Tagesstruktur, mögliche Ablenkungen sowie ein Motivationsmangel, der sich durch längere Phasen des Fernunterrichts zuspitzen kann (Pelikan et al. 2021; Schönbächler et al. 2020). So ist aus Schüler*innensicht der Wegfall eines geregelten Tagesablaufs mit festen Strukturen eine negative Konsequenz der Schulschließungen, da es für sie schwierig ist, ausreichend Selbstdisziplin und Motivation aufzubringen, um sich ihren Alltag selbst zu strukturieren (Huber et al. 2021, S. 114f.). Auch aus Perspektive der Lehrkräfte stellen eine fixe Tagesstruktur sowie die Fähigkeit der Schüler*innen zur Selbstorganisation zentrale Herausforderungen während des Fernunterrichts dar (Steiner et al. 2021, S. 45f.).

Neben allgemeinen Herausforderungen des Distance Learning wird insbesondere die Verschärfung bereits bestehender Bildungsungleichheiten aufgrund der Covid-bedingten Schulschließungen zuungunsten von Kindern aus sozial schwachen Verhältnissen kritisch diskutiert (Anger und Plünnecke 2021; Frohn 2020, 2021; Helm und Postlbauer 2021; Hammerstein et al. 2021; Huebener et al. 2021; Schwab und Lindner 2020). Eine wichtige Rolle wird hierbei den Digitalisierungsprozessen zugeschrieben, die im schulischen Kontext seit Beginn der Pandemie vorangetrieben werden. Von Bedeutung sind unterschiedliche Faktoren, wie die technische Ausstattung und digitalen Kompetenzen der Schüler*innen und ihrer Eltern (Helm et al. 2021a; Helm und Huber 2022; Kwik 2020; Senkbeil et al. 2019) sowie allgemein die elterlichen Unterstützungsleistungen (Holtgrewe et al. 2021; Sander et al. 2021; Steiner et al. 2021), die u. a. mit dem sozioökonomischen Status der Familie zusammenhängen.

Diese mit Digitalisierungsprozessen zusammenhängenden Ungleichheiten werden schon seit längerem im Kontext der Digital-Divide-Forschung diskutiert. Diese baut auf der These der digitalen Spaltung auf und drückt die Befürchtung aus, „dass sich im Zuge der unterschiedlichen Nutzung neuer Medien soziale Ungleichheiten verstärken“ (Zillien 2009, S. 82), wobei drei Forschungsstränge unterschieden werden: Der First(‑Level) Digital Divide (Attewell 2001; Hargittai 2002) beschreibt jene Unterschiede im digitalen Raum, die auf einer Differenzierung von Nutzer*innen und Nicht-Nutzer*innen basieren. Im Fokus steht die Frage nach Zugangsmöglichkeiten zu digitalen Geräten. Der Second(‑Level) Digital Divide rückt die unterschiedliche Nutzung des Internets in den Fokus, während der Third(‑Level) Digital Divide die Verwertbarkeit von Wissen aufgreift (van Deursen und Helsper 2015, S. 30). Im Fokus steht die Analyse von möglichen Vorteilen, die sich für Personen mit einem höheren sozioökonomischen Status durch die Art und Weise der Internetnutzung auch außerhalb des digitalen Raumes ergeben (Scheerder et al. 2017; van Deursen et al. 2017).

Neben zahlreichen Ansätzen und Modellen^2^ zur Erklärung und Analyse digitaler Ungleichheit erscheint für den Schulkontext insbesondere das von Senkbeil et al. (2019) verwendete Stufenmodell zur Beschreibung sozialer Ungleichheiten im Umgang mit digitalen Medien gewinnbringend. Im Rahmen der ICILS Studie 2018 werden Unterschiede von Schüler*innen im Hinblick auf folgende Dimensionen des Digital Divides näher analysiert: (1) materieller und physischer Zugang zu digitalen Geräten, Software sowie das Vorhandensein eines Internetzugangs; (2) motivationale Aspekte wie Einstellungen und Werthaltungen gegenüber dem Einsatz neuer digitaler Medien sowie die Motive für die Nutzung ebendieser; (3) die Nutzungshäufigkeit und -dauer digitaler Medien sowie deren Anwendungszwecke; und (4) die digitalen Kompetenzen, die für einen kompetenten Umgang mit digitalen Medien vorausgesetzt werden (Senkbeil et al. 2019, S. 303). Im Modell wird ein hierarchischer Zusammenhang zwischen den Dimensionen angenommen, wobei digitale Kompetenzen eigene Erfahrungen in der Nutzung und Anwendung digitaler Medien voraussetzen, denen wiederum grundlegende Werthaltungen gegenüber digitalen Medien vorangehen. Basis hierfür ist jedoch, dass ein Zugang zu digitalen Medien überhaupt erst vorhanden ist (van Deursen und van Dijk 2019; van Dijk 2005). Die von Senkbeil et al. (2019) systematisch analysierten Dimensionen digitaler Ungleichheit werden im vorliegenden Beitrag aufgegriffen und vor allem jene Aspekte thematisiert, die vor dem Hintergrund der Covid-19 Pandemie besonders relevant erscheinen – insbesondere (a) die technische Ausstattung der Schüler*innen im häuslichen Umfeld^3^ und (b) digitale Kompetenzen. Vor dem Hintergrund der Pandemie und den Anforderungen im Distance Learning erscheint es zudem notwendig, auch (c) die elterlichen Unterstützungsleistungen näher zu beleuchten (Holtgrewe et al. 2021; Steiner et al. 2021).

- Daten für Deutschland aus dem eGovernment Monitor 2021 zeigen, dass ein niedrigeres Einkommen mit geringerer *technischer Ausstattung* einhergeht. So stehen Kindern von einkommensstarken Familien durchschnittlich drei unterschiedliche Geräte zur Verfügung, die sie für schulische Zwecke nutzen können, während es in einkommensschwachen Familien durchschnittlich ein Gerät ist (Initiative D21 2021, S. 5). Für Österreich zeigt sich auf den ersten Blick eine gute technische Ausstattung der Schüler*innen. So verfügen über 95 % der Schüler*innen zuhause über einen Computer, den sie für schulische Tätigkeiten verwenden können (OECD 2020). Unterschiede zwischen den Schüler*innen im Hinblick auf ihre soziale Herkunft sind hierbei kaum festzustellen. Pessl und Steiner (2021) argumentieren allerdings, dass sich die technischen Anforderungen während der Phasen des Fernunterrichts erhöhen, „da potenziell mehrere Personen pro Haushalt im Home-Office und Distance-Schooling einen Computer nutzen mussten und darum die gleichzeitige Verfügbarkeit leichter an die Grenzen geraten konnte“ (Pessl und Steiner 2021, S. 187). Dadurch werde die digitale Kluft zwischen Schüler*innen aus sozialschwachen und vergleichsweise privilegierten Haushalten tendenziell unterschätzt. Dies bestätigt sich in einer Umfrage von Schober et al. (2020), die zeigen konnten, dass während des ersten Lockdowns ein Anteil von 16 % der Schüler*innen keinen eigenen Computer, Laptop oder ein Tablet zur Verfügung hatte, wobei dies vor allem Kinder aus nichtakademischen Haushalten betraf.
- Ackeren et al. (2020) weisen jedoch darauf hin, dass die technische Ausstattung lediglich ein Aspekt ist, den es zu berücksichtigen gilt. Auch die fehlenden *Kompetenzen* im Umgang mit digitalen Medien führen zu einer Verschärfung bereits bestehender Ungleichheiten. Im Rahmen der ICILS Studie 2018 konnte festgestellt werden, dass die computer- und informationsbezogenen Kompetenzen der Schüler*innen in Deutschland sehr stark mit dem kulturellen Kapital des Elternhauses zusammenhängen. So weisen Schüler*innen der achten Schulstufe aus Familien mit hohem kulturellen Kapital weitaus höhere digitale Kompetenzen auf als jene aus Familien mit niedrigem kulturellen Kapital. Insgesamt 43 % der befragten Schüler*innen aus Familien mit niedrigem kulturellen Kapital weisen lediglich rudimentäre digitale Kompetenzen auf (maximal Kompetenzstufe II) und verfügen über sehr einfache Anwendungskompetenzen (z. B. einen Link anklicken) und basale Wissensbestände hinsichtlich der Bearbeitung von Dokumenten (z. B. Suchfunktionen nutzen). Der Anteil von Schüler*innen aus Familien mit hohem kulturellen Kapital, die maximal Kompetenzstufe II erreichen, beläuft sich auf 19 % und fällt somit wesentlich geringer aus (Senkbeil 2019, S. 314).
  Im Hinblick auf die Ausstattung mit digitalen Geräten sowie die Nutzungskompetenzen zeigen González-Betancor et al. (2021), dass vor allem der Zugang zu technischen Geräten sehr stark mit dem sozioökonomischen Status der Herkunftsfamilie zusammenhängt. Nutzungshäufigkeit und -kompetenz sind hingegen wesentlich davon geprägt, wie Schulen digitale Medien nutzen und Lehrpersonen diese in ihren Unterricht integrieren. Dieser Aspekt überwiegt den Einfluss des sozioökonomischen Status, woraus die Autorinnen schließen, dass eine Einbindung von digitalen Medien im Schulkontext kompensatorisch auf soziale und damit zugleich digitale Ungleichheiten wirken kann. Auch Tawfik et al. (2016) kommen in einer Zusammenschau einer Reihe an Studien zum Ergebnis, dass der Einsatz digitaler Medien im schulischen Kontext ungleichheitsmindernde Effekte haben kann; umgekehrt können aber auch bestehende Lücken vergrößert werden. Umso mehr scheint nötig, pädagogische Konzepte und Digitalisierungsstrategien zu entwickeln, die eine erfolgreiche Implementierung von digitalen Medien in der Schule ermöglichen. Dass derartige Konzepte und Strategien fehlen und die Schule das pädagogische Potenzial der Digitalisierung verkenne, wurde bereits vor Beginn der Pandemie kritisiert (Schmid et al. 2017
- Neben der technischen Ausstattung und den digitalen Kompetenzen spielen auch die *elterlichen Unterstützungsleistungen* eine wesentliche Rolle beim Distance Learning. Dies ist darauf zurückzuführen, dass dabei das Lernen in die private Wohnumgebung der Schüler*innen verschoben wird und so die elterliche Unterstützung bei Hausübungen und beim Lernen wesentlich dazu beiträgt, dass Schüler*innen die an sie gestellten schulischen Anforderungen erfüllen können. Studien aus der Zeit der Schulschließungen im Frühjahr 2020 zeigen, dass ein Anteil von 21 % der Schüler*innen zuhause keine bzw. nicht die notwendige Unterstützung beim Lernen erhielt (Schober et al. 2020). Eine Studie von Refle et al. (2020, S. 30f.) bemisst diesen Anteil sogar auf ein Drittel der Schüler*innen. Das Fehlen der elterlichen Unterstützung ist auch einer Lehrer*innenbefragung von Steiner et al. (2021) zufolge ein Kernproblem im Distance Learning. Dass Eltern ihre Kinder nicht in dem Maße unterstützen können, wie diese es im Corona-bedingten Distance Learning benötigen würde, liegt laut Studien, die Eltern befragt haben, vorwiegend an fehlenden zeitlichen Ressourcen (für einen Überblick siehe Helm et al. 2021a, S. 272f.). Des Weiteren sind auch die fachlichen Schwierigkeiten der Eltern umso höher, je niedriger deren formale Bildung ist. In einer Schülerbefragung geben 60 % der Schüler*innen mit hoch qualifizierten Eltern an, dass ihre Eltern sie fachlich und inhaltlich unterstützen konnten. Bei Kindern mit einfach und mittel qualifizierten Eltern beläuft sich der Anteil auf gut ein Drittel (Holtgrewe et al. 2021, S. 18).

Dass diese Benachteiligungen nicht auf die Dauer des Distance Learning begrenzt bleiben, zeigen Daten der deutschen SOEP-CoV-Studie (Zinn und Bayer 2021). Demnach reduzierte sich vor allem bei Familien mit niedrigem Bildungsniveau das zeitliche Ausmaß der elterlichen Unterstützung unmittelbar nach Phasen der Schulschließungen im Frühjahr und Winter 2020. Während die Lern- und Schularbeitszeiten der Kinder während der Phasen des Fernunterrichts unabhängig vom Bildungsgrad der Eltern ähnlich ausfielen, zeigt sich insbesondere in den ersten Wochen nach den Lockdowns ein Auseinanderklaffen der Lernzeiten um gut eine Stunde zuungunsten von Kindern aus bildungsfernen Haushalten. Den frühen Rückzug der elterlichen Unterstützung führen die Autor*innen auf Überforderung, Erschöpfung und eine schnelle Rückübertragung der Verantwortung für die Beschulung der Kinder an die Schulen zurück (Zinn und Bayer 2021, S. 4).

## Methodik

Die im Folgenden präsentierten Ergebnisse beruhen auf einer Interviewstudie mit insgesamt 71 qualitativen, leitfadengestützten Interviews, die im Rahmen des an der Pädagogischen Hochschule Steiermark durchgeführten Projektes „Schule nach Corona!?“ zwischen Herbst 2020 und Sommer 2021 in der Steiermark geführt wurden. Die Interviews fokussierten drei Akteursgruppen: Erstens Lehrer*innen und Schulleitungen (Primarstufe und Sekundarstufe I/II; 24 Interviews^4^), zweitens Mitarbeiter*innen aus dem Bereich der psychosozialen Unterstützungssysteme an Schulen (33 Interviews)^5^. Hinzu kommen drittens 14 Interviews (zumeist Gruppeninterviews) mit insgesamt 48 Schüler*innen zwischen sechs und 15 Jahren^6^. Das Sampling erfolgte vor allem im Hinblick darauf, einen differenzierten Blick auf Bildungsungleichheiten in Pandemiezeiten aus verschiedenen Perspektiven zu erhalten. Daher wurde einerseits außerschulischen Akteursgruppen ein großer Anteil eingeräumt, andererseits wurde darauf geachtet, im Hinblick auf Schultypen und Schulstandorte (städtisch vs. ländlich; unterschiedliche sozialstrukturelle Zusammensetzung der Schüler*innenschaft) eine große Bandbreite abzudecken. Die Interviews wurden transkribiert; die Auswertung orientierte sich an der inhaltlich strukturierenden qualitativen Inhaltsanalyse nach Kuckartz (2012). Das Codesystem dafür wurde schrittweise aus dem Material selbst gewonnen und in mehreren Reflexionsstufen überarbeitet. In der Folge wird bei wörtlichen Zitaten auf die Interviews in Form von kurzen Siglen (z. B. „Lehrerin_01_VS“, „Schulsozialarbeit_01“) verwiesen.

## Ergebnisse I: Distance Learning und digitaler Unterricht

Wie wurde seit Beginn der Covid-19 Pandemie der Unterricht in der Distanz und unter Verwendung digitaler Medien gestaltet? Diese Frage wird anhand der Daten aus den geführten Interviews adressiert, wobei auf Herausforderungen und Handlungsstrategien auf Seiten von Lehrer*innen und Schüler*innen eingegangen wird.

### Handlungsstrategien von Lehrer*innen im digitalen Unterricht: Holprige Anfänge – erfolgreiche Implementierung?

Die Lehrer*innen in den Interviews berichteten in Bezug auf die Gestaltung des Unterrichts und die Organisation des Distance Learning von verschiedenen Lernprozessen, denen sie als Lehrer*in bzw. als Schule begegnen mussten. Dass die Umstellung auf Distance Learning vor allem im ersten Lockdown überraschend und plötzlich kam, wurde in einer Reihe an Interviews thematisiert (u. a. Lehrer_04_AHS, 23; Lehrer_06_MS, 15; Lehrerin_13_VS, 57). Die konkrete Umsetzung gestaltete sich je nach Schultyp, teils auch je nach Standort, unterschiedlich. Insgesamt wurde im Bereich der Primarstufe häufiger mit analogen „Lernpaketen“ gearbeitet, während digitale Medien und Lernangebote erst ab der Sekundarstufe eine zentrale Rolle spielten. Dabei wird die Umsetzung gerade anfangs häufig als chaotisch wahrgenommen – aus der Außenperspektive der Lernhilfe ergab sich etwa das folgende Bild:

In den NMSen gab’s halt wirklich eine Vielzahl an Lehrern, eine Vielzahl an unterschiedlichen Plattformen – ein Lehrer hat etwa Padlet verwendet, einer schickte Aufgaben per Mail, einer wollte Microsoft Teams verwenden, wofür man wieder einen PC gebraucht hätte, was nicht alle hatten; manche schickten Aufgaben zum Ausdrucken, bei anderen hätte man einen Link gleich online bearbeiten müssen […]. (Lernhilfe_01, 34)

Auf der anderen Seite ist aber auch die Rede von einer „positiven Spannung“ und „Neugierde“ (Lehrerin_20_AHS, 2), die Möglichkeiten digitaler Medien in der Schule einmal stärker auszuprobieren, und von einer Bereitschaft zu Fortbildungen im Bereich digitaler Medien (Lehrerin_12_VS, 46; Schulleitung_03_VS, 12). In den Interviews wird aber auch thematisiert, dass manche Lehrer*innen wenig Bereitschaft zeigten, digitale Möglichkeiten zu nutzen (Schulsozialarbeit_07, 43; Lehrer_06_MS, 51). Insbesondere in den Interviews mit den Schüler*innen kommt zur Sprache, dass den Lehrer*innen selbst teils die digitalen Kompetenzen fehlen:

Also die älteren Lehrer sind auf jeden Fall nicht wirklich damit [mit der Umstellung auf digitalen Unterricht] klargekommen. […] Die haben halt immer uns gefragt ‚Wie macht man das?‘ und ‚Wie macht man das?‘ (KiJu_Gruppe03_JuZ, 180–188)

Insgesamt lässt sich jedoch anhand der Interviews für den Verlauf des Schuljahres 2020/21 eine weitgehende Konsolidierung des Unterrichts im Distance Learning mit digitalen Methoden sowie eine sukzessive Vereinheitlichung und Verbesserung der Handhabung digitaler Lehre feststellen. Vor allem ab der Sekundarstufe wurde die Verwendung von Microsoft Teams und die Abhaltung von Online-Stunden den interviewten Lehrer*innen zufolge zum Standard (u. a. Lehrer_02_AHS, 70; Lehrerin_20_AHS, 4). Auf Ebene der Volksschulen blieb die Einbindung digitaler Mittel eher fakultativ bzw. auf einzelne Tools beschränkt (u. a. Lehrerin_13_VS, 59; Lehrerin_11_VS, 16); dafür berichten gerade auf dieser Ebene Lehrer*innen von einer positiven Bereicherung, von „Spaß“ und Austausch im „virtuellen Klassenzimmer“ (Lehrerin_15_VS, 37), wo das Miteinander im Distance Learning gut funktioniert habe. Von Lehrer*innen höherer Schulstufen – die insgesamt wesentlich längere Zeit auf Distance Learning angewiesen waren – werden stärker auch Probleme angesprochen. Dies betrifft Lücken in der technischen Ausstattung und fehlende digitale Kompetenzen, aber auch die Interaktionssituation im Unterricht: Mehrere Lehrer*innen berichten, dass ihrem Eindruck nach die Kinder und Jugendlichen gerade bei längeren Online-Einheiten Schwierigkeiten hätten, inhaltlich zu folgen und sie kaum in der Lage seien, über die gesamte Einheit hinweg aufmerksam und konzentriert zu sein (Lehrerin_08_MS, 11): „Man war dort, aber es fand kein Lernen statt bei 27 [Schüler*innen] vor der Kamera“ (Lehrerin_20_AHS, 36). Auch in den Interviews mit den Schüler*innen wurde deutlich, dass die Fokussierung der Aufmerksamkeit auf den Unterricht im Distance Learning häufig nicht gelang. So berichten sie etwa davon, dass sie, während am Handy oder Laptop der Online-Unterricht gelaufen sei, nebenher ferngesehen, gespielt oder gegessen hätten oder sogar wieder eingeschlafen seien (KiJU_Gruppe06_JuZ, 54; KiJU_Gruppe05_JuZ/, 90; KiJu_Gruppe03_JuZ, 27). Vor diesem Hintergrund stellt sich für die Lehrer*innen im digitalen Unterricht – sei es über Online-Stunden, sei es über Arbeitsaufträge – die zentrale Herausforderung, pädagogische Konzepte zu entwerfen, die den Einsatz digitaler Medien entsprechend didaktisch modellieren. Das bedeutet, nicht nur Digitalisierung des Unterrichts ‚an der Oberfläche‘ zu betreiben, sondern die „Tiefenstrukturen“ als Grundlagen von Unterrichtsqualität – insbesondere kognitive Aktivierung und unterstützende Strukturierung – auch bei der Nutzung digitaler Unterrichtsmittel sicherzustellen (Voss und Wittwer 2020, S. 605f.). Solche Bemühungen werden in den Interviews zwar durchaus von einigen Lehrer*innen berichtet, aber nur von einigen als Erfolg beschrieben (u. a. Lehrer_04_AHS, 47), während bei anderen vor allem das Gefühl des Scheiterns vorherrscht (Lehrerin_08_MS, 11). Dies gerade dort, wo in Bezug auf die eigene Schüler*innenschaft auch mangelnde digitale oder sprachliche Kompetenzen und fehlende elterliche Unterstützung konstatiert werden (Lehrerin_03_MS, 13 und 25; Beratungslehrerin_02, 63).

### Schüler*innen: Motivation und Strukturierung als zentrale Herausforderungen im Distance Learning

Die Lernerfahrungen im Distance Learning werden in den Interviews für einen Teil der Schüler*innen als durchaus positiv beschrieben. Manche seien „regelrecht aufgeblüht“ (Lehrerin_20_AHS, 14) und das Lernen zuhause sei ihnen leichtgefallen, weil sie in Ruhe konzentriert arbeiten konnten (Lehrer_02_AHS, 51; Schulsozialarbeit_07, 43). Zu sehen, was sie alleine schaffen können, habe diesen Kindern zu Selbstständigkeit wie auch zu Selbstbewusstsein verholfen (u. a. Lehrer_06_MS, 53; Lernhilfe_03, 22; Lehrerin_09_VS, 48). Auf der anderen Seite stehen Berichte über eine Vielzahl an Herausforderungen, die sich vor allem mit Lernmotivation und -strukturierung in Verbindung bringen lassen – also ähnliche Themenbereiche wie jene, die bereits oben im Hinblick auf die Lehrer*innen-Perspektive auf Unterrichtsgestaltung im Distance Learning als zentral herausgearbeitet wurden.

Dass *Lernmotivation* in den Phasen des Fernunterrichts eine Herausforderung sei, wird im Großteil der geführten Interviews thematisiert. Lehrer*innen wie auch Mitarbeiter*innen der Unterstützungssysteme berichten dabei, dass die Lernmotivation mit zunehmender Dauer des Distance Learning abgenommen habe. Dementsprechend seien es vor allem die Schüler*innen der weiterführenden Schulen (Sekundarstufe II) – welche von Anfang November 2020 bis Mitte Mai 2021 keinen Präsenzunterricht hatten – die im Verlauf des Schuljahres, vor allem in der Zeit nach Weihnachten, zunehmend „verfallen“ bzw. „weggebrochen“ seien (Lehrerin_18_AHS, 9). Je länger es gedauert habe, desto mühseliger sei es geworden – viele konnten sich nicht mehr aufraffen, zum Unterricht aufzustehen oder schliefen während der Stunde wieder ein, wie die Lehrerin einer berufsbildenden höheren Schule berichtet (Lehrerin_19_BHS, 8). Eine Lehrerin im Gymnasium schildert die zunehmend schwierige Lernsituation, die sich ihrer Meinung nach bei einigen Schüler*innen schon zu einer depressiven Verstimmung zuspitzt, folgendermaßen:

Die Großen haben mir oft gesagt ‚Es tut mir so leid, ich kenne das selber nicht, wie ich bin. Es tut mir so leid, Frau Professor‘, sagen sie immer. […] ‚Ich schaffe das nicht, dass ich mich hinsetze.‘ (Lehrerin_20_AHS, 56)

Aber auch von Schüler*innen der Mittelschulen und gymnasialen Unterstufen wird berichtet, dass sie am Ende der Distance-Learning-Phasen nur mehr schwer zu motivieren waren: „Da ist jetzt einfach die Luft draußen“ (Lernhilfe_03, 18).

Diese Statements verweisen bereits darauf, dass die Corona-bedingten Einschränkungen in der Schule Fähigkeiten erfordern, sich selbst für das Lernen zu begeistern und zu motivieren. Mehrmals fällt hier in den geführten Interviews das Schlagwort, dass die Kinder zunehmend Aufträge „abarbeiteten“ (Lehrerin_08_MS, 5; Schulleitung_03_VS, 46) und nur genau das täten, was ihnen aufgetragen wurde. Lernen erfolge nicht mehr aus eigenem Antrieb, wobei diese Einstellung zum Lernen nicht erst im Distance Learning entstanden sei, in diesem Kontext aber besonders zum Tragen komme (Lehrerin_20_AHS, 18). Verstärkend kommen die im Distance Learning und auch im von Corona-Maßnahmen bestimmten Präsenzbetrieb mangelnde Freude und das fehlende soziale Miteinander hinzu: Das Lernen wird von den Interviewpartner*innen als „zäh“ beschrieben (Lehrerin_15_VS, 24) – das „Dazwischen“, das Miteinander und die „Leichtigkeit“ hätten gefehlt (Schulleitung_03_VS, 42). Auch die Interaktion mit den Mitschüler*innen, das gegenseitige Motivieren oder Erinnern an Aufgaben fielen in den Phasen der Schulschließung weg (Lehrerin_19_BHS, 10; KiJu_Gruppe11_VS, 50).

Die Schulschließungen weichen zudem auch die *Strukturen*, die durch die Schule vorgegeben werden, in großem Ausmaß auf. Dass der Unterricht in der Früh beginnt, dass es eine bestimmte Zeit des Tages gibt, die zum Lernen vorgesehen ist – all das wird im Lockdown relativiert. Zwar meinen viele der interviewten Schüler*innen, dass sie die freiere Zeiteinteilung genossen hätten – vor allem das Ausschlafen, aber auch die Möglichkeit, jederzeit eine Pause machen und sich entspannen zu können (KiJu_Gruppe04_JuZ, 52; KiJu_Gruppe11_VS, 48–51). Auf der anderen Seite stehen jedoch Erzählungen von vielen Schüler*innen, die davon berichten, wie schwer es für sie war, sich zum Lernen aufzuraffen und sich nicht ablenken zu lassen: „Wenn Aufträge kommen, sagt man, dass man es später macht. Aber man will es gar nicht machen“ (KiJU_Gruppe07_JuZ, 48). Schüler*innen berichten auch von Überforderung, sich die Arbeit so einzuteilen, dass bis zum Abgabetermin alles erledigt ist, von großem Stress und vom Gefühl, dabei auf sich allein gestellt zu sein (KiJu_Gruppe11_VS, 53). Je stärker diese Überforderung, desto eher gehen die Schüler*innen dazu über, die Aufgaben gar nicht mehr zu machen (Lernhilfe_02, 9): „Corona-Paket! […] Ja … da hab ich nix gemacht. […] Ich hab mich nicht ausgekannt“ (KiJu_Gruppe02_JuZ, 72ff.).

Ob bzw. inwieweit Schüler*innen sich auch nach längeren Phasen des Distance Learning zum Lernen motivieren können und es schaffen, selbstständig ihren Tag und ihre Aufgaben zu strukturieren, hängt von unterschiedlichen *Faktoren* ab. Selbstständigkeit und Lernmotivation im Distance Learning hängen zunächst (mehr als im Präsenzbetrieb) davon ab, inwiefern Schüler*innen auf ein Grundverständnis aufbauen können, um die gestellten Aufgaben bearbeiten zu können – es spielen also bestehende Leistungsunterschiede eine Rolle. Und das bedeutet umgekehrt, dass jene Schüler*innen, die sich schon in Präsenz schwertun, ihre Aufträge zu erledigen, sich im Distance Learning noch schwerer tun (Lehrerin_05_VS, 69; Lehrer_04_AHS, 53; Lernhilfe_02, 7): „Man hat oft das Gefühl, sie wissen nicht, wo oben und unten ist“ (Beratungslehrerin_02, 69), wird es in einem Interview ausgedrückt. Daneben hängt die Fähigkeit zur Strukturierung und zum selbstständigen Lernen auch in großem Ausmaß von bereits erlernten, verfügbaren Lernstrategien ab: In Bezug auf ihre Klasse weist eine Lehrerin im Interview darauf hin, dass sich die Schüler*innen schwertun, weil sie die nun geforderte Art von „Lernen“ nie „gelernt haben“ (Lehrerin_08_MS, 17); von anderen Lehrer*innen wird dagegen durchaus berichtet, dass ihre Schüler*innen auch deshalb gut mit dem Distance Learning zurechtkamen, weil sie selbstorganisiertes und offenes Lernen bereits gewohnt gewesen seien (Lehrerin_11_VS, 16; Lehrerin_14_VS, 5). Dabei werden gerade aus dem Primarbereich durchaus positive Erfahrungen mit dem offenen Lernen auf Grundlage von Arbeitsplänen im Distance Learning berichtet; dagegen sei gerade in der Sekundarstufe I von Kindern oft mehr Selbstständigkeit erwartet worden, als diese einbringen konnten (Lehrerin_16_VS, 49). Auch hier spielen letztlich einerseits eine gelungene Gestaltung der konkreten Unterrichtsmittel eine entscheidende Rolle, andererseits aber auch die ungleichen Bedingungen, unter denen Schüler*innen im Distance Learning selbstständig arbeiten. Diese werden im Folgenden näher beschrieben.

## Ergebnisse II: Facetten digitaler Ungleichheiten in der Pandemie

Wie hängen nun die dargestellten Strategien und Herausforderungen von Distance Learning und digitalisiertem Lernen mit Ungleichheiten zusammen? Diese Frage wird im Folgenden anhand drei zentraler Aspekte behandelt: In Anlehnung an Senkbeil et al. (2019, S. 303) werden die technische Ausstattung der Schüler*innen im häuslichen Umfeld und deren digitale Kompetenzen näher beleuchtet sowie ergänzend auf die elterliche Unterstützung eingegangen, die vor dem Hintergrund einer Verlagerung des Lernens in den häuslichen Kontext während der Covid-19 Pandemie besonders relevant erscheint (Holtgrewe et al. 2021; Steiner et al. 2021).

### Technische Ausstattung der Schüler*innen im häuslichen Umfeld

Die größten Schwierigkeiten mit der Ausstattung der Schüler*innen in Bezug auf die technischen Voraussetzungen werden aus dem ersten Lockdown im Frühjahr 2020 berichtet. Die Covid-bedingte Umstellung auf Distance Learning führte in vielen Fällen erstmalig den Schulen bzw. Lehrer*innen vor Augen, welche Mängel es in der technischen Ausstattung der Schüler*innen gibt: „Ich glaube, da haben viele Lehrer nicht damit gerechnet, dass es in Österreich immer noch viele Leute gibt, die nicht technisch ausgestattet sind“ (Flexible Hilfen_03, 12). Dass also Kinder keinen Laptop zur Verfügung haben oder sich ein Gerät mit den Eltern und Geschwistern teilen müssen; dass viele keine Druckmöglichkeit zuhause haben oder keinen Internetzugang (u. a. Flexible Hilfen_03, 12; Schulsozialarbeit_02, 15; Jugendcoaching_02, 10). Berichtet wird etwa, dass sie von den Lehrer*innen teilweise mit ihrem Handy zu öffentlichen WLAN-Hotspots geschickt werden:

McDonalds, der hat einen Hotspot, den hat er, Gott sei Dank, auch eingeschaltet gelassen im ersten Lockdown. Im zweiten Lockdown war das deutlich schwieriger, weil es war November und da kannst du den Schülern nicht sagen, dass sie den ganzen Tag neben dem McDonalds im Freien verbringen. (Lehrerin_19_BHS, 8)

Im weiteren Verlauf der Pandemie zeichnet sich insbesondere im Hinblick auf die technische Ausstattung in vielen Fällen eine Verbesserung ab. In den Interviews wird davon berichtet, dass sich Familien angesichts der veränderten Relevanz von technischer Ausstattung selbst um Geräte und bessere Internetverbindungen kümmerten (Flexible Hilfen_01, 40); dazu werden Sammelaktionen und Spendenaufrufe durch diverse Hilfsorganisationen angesprochen (u. a. Lernhilfe_01, 20; Schulsozialarbeit_02, 17), aber auch dass Schulen selbst aktiv wurden und Geräte für Kinder bereitstellten (Lehrerin_03_MS, 79; Schulsozialarbeit_07, 35). Dennoch wird auch aus den im Laufe des Schuljahres 2020/21 geführten Interviews deutlich, dass vieles nach wie vor fehlt – und dass die Voraussetzungen von Schüler*innen, am Distance Learning teilzunehmen, ungleich bleiben. Dass Geräte mangelhaft funktionierten oder mit Geschwistern und Eltern geteilt werden mussten, wurde in den Interviews mehrmals berichtet (KiJu_Gruppe02_JuZ, 62; Lehrerin_01_VS, 50; Jugendcoaching_02, 15) – aber auch, dass Familien Leihgeräte teilweise selbst ablehnten, etwa aus Sorge davor, dass diese beschädigt werden (Lernhilfe_01, 20).

Diese bestehenden Ungleichheiten werden jedoch keineswegs an allen Schulen so wahrgenommen bzw. berücksichtigt. In einem zur Zeit des Lockdowns im Winter 2020 geführten Interview berichtet eine Volksschullehrerin etwa: Wer von den Schüler*innen keinen Laptop mit Internetverbindung habe, könne an den wöchentlichen Erarbeitungsstunden eben nicht teilnehmen (Lehrerin_07_VS, 39). Die daraus resultierenden Probleme für jene Schüler*innen, die dadurch von den gemeinsamen Online-Stunden ausgeschlossen werden, werden von der Lehrerin ausgeklammert. Eine Lehrerin aus einer anderen Schule meint in diesem Zusammenhang: „Es ist auch nicht hinterfragt worden, kann jedes Kind irgendwie auf diese Online-Pinwand zugreifen? Es ist irgendwie: Ja, entweder kann es das, oder wenn nicht, ist halt blöd gelaufen“ (Lehrerin_13_VS, 79).

### Digitale Kompetenzen

Dass manche Schüler*innen von sich aus darauf verzichten, Leihgeräte anzunehmen, verweist bei genauerem Hinsehen auch auf mangelnde Nutzungskompetenzen, die Schüler*innen den Umgang mit PC oder Laptop erschweren. Er arbeite „lieber auf dem Handy. […] Handy ist leicht“, meint dazu ein Schüler im Interview (KiJU_Gruppe07_JuZ, 31). Wenn sich Schüler*innen also von den Anforderungen, die das Bedienen eines PCs stellt, überfordert fühlen, weichen sie auf das ‚gewohnte‘ Handy aus – auch wenn es bedeutet, dass sie dem Online-Unterricht schlechter folgen können. Dass es derart gravierende Lücken bei den digitalen Kompetenzen der Schüler*innen gibt, war ebenfalls überraschend für viele der interviewten Lehrer*innen und Mitarbeiter*innen psychosozialer Unterstützungssysteme:

Alle Jugendlichen haben zwar ein Handy, ein Smartphone, sind eigentlich gut ausgestattet, aber können damit nicht umgehen. […] Sie haben sich da teilweise relativ schwergetan, außer telefonieren und WhatsApp, das Handy auch anders zu nutzen. (Jugendcoaching_02, 10)

Auch hier kann im Verlauf der Pandemie mit den Phasen des Distance Learning und der größeren Relevanz des Themas eine Verbesserung und Ausweitung von Kompetenzen für viele festgestellt werden. Dennoch bleiben bestimmte Herausforderungen bestehen, die mit strukturellen Ungleichheiten verknüpft sind – und zwar nicht nur mit dem ökonomischen Hintergrund, sondern auch mit dem kulturellen Kapital und den sprachlichen und medialen Kompetenzen, die Schüler*innen mitbringen. Dabei haben die persistierenden digitalen Ungleichheiten auch Auswirkungen auf den Unterricht, weil die Gestaltungsmöglichkeiten der Lehrer*innen durch die Ausstattung und Kompetenzen der Schüler*innen mitbestimmt werden. Eine Beratungslehrerin beschreibt dies anhand zweier unterschiedlicher Volksschulen, an denen sie tätig ist:

Die [Schule], wo die Diversität viel höher ist und viele Sprachen-Nachteile usw. sind: Die sind dann vielleicht doch im klassisch analogen Sinn beschult worden. In der anderen Schule hat man viel mehr Medien noch dazugetan. Also wo Lernvideos raufgestellt wurden, wo wirklich jeden zweiten Tag oder sogar jeden Tag eine Onlineeinheit stattfand. Also das hat in der anderen Schule quasi wenig Sinn, weil die auch einfach so schlecht ausgestattet sind zuhause. (Beratungslehrerin_02, 49)

Die Ergebnisse legen in diesem Zusammenhang nahe, dass digitale Ungleichheiten während der Distance-Learning-Phasen tendenziell weitergeführt bzw. verstärkt werden – und zwar schon auf der Primarstufe, gerade weil digitale Kompetenzen hier noch als ‚fakultativ‘ erachtet werden: Kinder mit guter Ausstattung bekommen teils bereits auf dieser Ebene mehr digitale Lernmöglichkeiten und können ihre Kompetenzen weiter ausbauen, während andere ihre fehlenden Kompetenzen nicht aufholen, da ihnen gar keine entsprechenden Angebote gemacht werden (können).

### Familiäre Unterstützungsleistungen

Ungleich sind auch die Voraussetzungen, die Schüler*innen für das Lernen zuhause vorfinden. Das Distance Learning führe in vielen Familien zu einer fachlichen wie auch psychischen Überforderung der Eltern, wurde in den Interviews oftmals berichtet (Schulsozialarbeit_07, 31; Lernhilfe_02, 17–19; Lehrerin_13_VS, 21; Beratungslehrer_03, 31): „Weil man ist auf einmal Mama, Lehrer, alles in einem gewesen. Manche haben vielleicht noch Homeoffice nebenbei. Das war für manche sicher sehr, sehr stressig“ (Lehrerin_15_VS, 36). Besonders prekär werden die Bedingungen vor allem für jene Kinder und Jugendliche beschrieben, die selbst mit Mehrfachbelastungen konfrontiert werden. So wird berichtet, dass manche ältere Jugendliche auch für andere Geschwister und Familienmitglieder zuständig gewesen und teils mit der Organisation des ganzen Haushalts betraut worden seien, während für das eigene Lernen nur mehr in der Nacht Zeit geblieben und eigene Freizeit gar nicht mehr vorhanden gewesen sei – eine starke Belastung, die auch zu psychischen Problemen geführt habe (Lehrerin_19_BHS, 6 und 40; Jugendcoaching_01, 19; Jugendzentren_02, 41).

Einen großen Einfluss haben familiäre Unterstützungsleistungen in den Phasen der Schulschließungen auch auf das Lernen selbst. Hier geht es nicht nur um Wissensvermittlung und -vertiefung^7^, sondern auch um Lernorganisation und -motivation: Nicht alle Eltern, so wird in den Interviews von Lehrer*innen und Mitarbeiter*innen in psychosozialen Unterstützungssystemen berichtet, hätten Verständnis dafür, dass auch während der Phasen des Distance Learning ein strukturierter Alltag eingehalten werden solle. Manche könnten zudem trotz Nachfragens und Nachhakens vonseiten der Schule nicht dafür sorgen, dass ihre Kinder die Aufgaben zeitgerecht erledigten (Lehrerin_20_AHS, 18; Lehrerin_03_MS, 67; Schulsozialarbeit_07, 3).

Damit Eltern oder andere Familienmitglieder Schüler*innen beim Distance Learning gut unterstützen können, bedarf es aber auch sprachlicher und medialer Kompetenzen. Der Wegfall der Präsenz und damit von nonverbalen Kommunikationsmitteln wie Gestik und Mimik könne, wie in den Interviews berichtet wird, vor allem für Personen mit schlechten Deutschkenntnissen eine besondere Herausforderung darstellen (Lehrerin_03_MS, 19; Lernhilfe_01, 44; Flexible Hilfen_02, 22). Zwar wird in den Interviews durchaus von Möglichkeiten berichtet, digitale Tools produktiv einzusetzen, indem etwa Nachrichten an Eltern in deren Erstsprache übersetzt werden (Schulsozialarbeit_07, 17) – die Ressourcen dafür stehen allerdings nur an einigen Schulen zur Verfügung. An anderen wird die Kommunikation mit Eltern, die kaum oder gar keine Deutschkenntnisse haben, in Zeiten des Distance Learning als besonders prekär beschrieben (Flexible Hilfen_03, 64). In den Interviews wird auch angesprochen, dass die teilweise komplexen schriftlichen Anweisungen, die auf den Lernplattformen gestellt wurden, generell für Personen überfordernd seien, die z. B. Probleme beim sinnerfassenden Lesen hätten oder mit schulischen Abläufen wenig vertraut seien: Die „verschachtelten Anweisungen“ hätten viele Kinder verwirrt, seien aber auch nicht „fassbar für viele Eltern“ gewesen (Lernhilfe_02, 9).

## Fazit

Der vorliegende Beitrag widmet sich der Fragestellung, welche Veränderungen und Entwicklungen die Covid-bedingten Umstellungen auf Distance Learning im Hinblick auf digitalen Unterricht an österreichischen Schulen mit sich brachten und wo in diesem Zusammenhang Ungleichheiten eine Rolle spielen. Dazu werden Daten einer multiperspektivischen qualitativen Interviewstudie herangezogen, die in der Steiermark in der Zeit zwischen Herbst 2020 und Frühjahr 2021 durchgeführt wurde.

Die Ergebnisse zeigen, dass die Covid-19 Pandemie in den meisten Schulen der Sekundarstufen eine rasche und weitreichende Einbindung von digitalen Formen des Unterrichtens und Lernens in den Phasen der Schulschließung mit sich brachte. Dies führte zu einem Ausbau an technischer Ausstattung sowie digitalen Kompetenzen – sowohl Lehrer*innen und Schule als auch Schüler*innen und die Lernumgebung zuhause betreffend. Was mit dem ersten Covid-bedingten Lockdown im März 2020 als überraschende, chaotische Umstellung begann, führte zu einer schrittweisen Konsolidierung und Einübung von Formen digitalen Unterrichtens und Lernens.

Dennoch zeigen die Ergebnisse der vorliegenden Studie deutlich, dass strukturelle Ungleichheiten im Hinblick auf verschiedene Dimensionen digitaler Ungleichheit weiterhin bestehen. Auf Ebene der technischen Ausstattung kann weiterhin nicht davon ausgegangen werden, dass alle Schüler*innen zuhause mit Geräten oder angemessenem Internetzugang ausgestattet sind. Aber auch in Bezug auf digitale Kompetenzen wird von großen Unterschieden berichtet. Dabei weisen die Ergebnisse darauf hin, dass Ungleichheiten durchaus vielschichtig miteinander verflochten sind. So zeigt sich etwa anhand der hier vorgestellten Daten, dass fehlende Kompetenzen auch zu Zurückhaltung bezüglich der Ausstattung führen können: Wer Sorge hat, sich mit dem Laptop nicht auszukennen, lehnt auch diesbezügliche Leihangebote ab und arbeitet weiterhin mit dem Handy, mit dem der Umgang bereits gewohnt und ‚leicht‘ ist. Die präsentierten Ergebnisse zeigen zudem eine Tendenz zur Verstärkung bestehender digitaler Ungleichheiten von Seiten der Schulen – insbesondere auf Ebene der Primarstufe, wo das Thema eher als fakultativer Zusatz wahrgenommen wird: Strukturell benachteiligten Schüler*innen werden etwa von Lehrer*innen tendenziell weniger digitale Angebote gemacht oder es wird hingenommen, dass sie an digitalen Unterrichtseinheiten nicht teilnehmen können. Die Ergebnisse legen in diesem Zusammenhang nahe, dass diese Kinder damit auch weniger Gelegenheiten haben, digitale Kompetenzen aufzubauen.

Es lässt sich festhalten, dass sich jene Faktoren, die bereits vor der Pandemie für die tiefgreifenden Ungleichheiten im österreichischen Bildungssystem ausschlaggebend waren – sozialer Status, ökonomische Voraussetzungen, Bildungsstand der Eltern und kulturelles Kapital – auch im Bereich digitalen Lernens weiter fortschreiben. Eine Weiterführung bzw. Verschärfung von Bildungsungleichheiten, wie sie in anderen wissenschaftlichen Studien diskutiert und mit digitalen Ungleichheiten in Zusammenhang gebracht wird (Anger und Plünnecke 2021; Helm et al. 2021a, b; Helm und Huber 2022; Pessl und Steiner 2021; Steiner et al. 2021), zeigt sich auch anhand der hier vorgestellten empirischen Ergebnisse. Als Grundproblem lässt sich dabei ausmachen, dass flächendeckende Digitalisierungsprozesse im schulischen Kontext erst durch die Pandemie initiiert wurden und diese Prozesse folglich als „Digitalisierung mit der Brechstange“ (Lehrer_06_MS, 51) wahrgenommen werden, wie es in einem Interview bezeichnet wurde. In den Vordergrund rücken damit Faktoren wie technische Ausstattung, digitale Kompetenzen und Unterstützungsleistungen, die die Schüler*innen *zuhause* bereits zur Verfügung hatten – oder eben nicht. Für eine gezielte Implementierung digitalen Unterrichtens, bei dem zuerst Fragen der Ausstattung geklärt, dann Kompetenzen aufgebaut und erst in einem dritten Schritt Unterrichtsinhalte über digitale Kanäle vermittelt werden, fehlten die zeitlichen und technischen Ressourcen. Fragen nach lernförderlichen Bedingungen im Distance Learning unter Einsatz digitaler Medien konnten, wenn überhaupt, erst nachgelagert bearbeitet werden. Die Potenziale digitaler Methoden in der Schule, die durchaus auch ungleichheitsmindernde Effekte haben können (González-Betancor et al. 2021; Tawfik et al. 2016), konnten in der Pandemie vor diesem Hintergrund nicht genutzt werden.

Als Handlungsempfehlungen, die sich auf Grundlage der hier präsentierten Ergebnisse formulieren lassen, lässt sich erstens festhalten, dass die im Zuge der Pandemie erreichten digitalen ‚Fortschritte‘ im schulischen Bereich weiterhin kritisch zu überprüfen und strukturelle Benachteiligungen zu berücksichtigen sind, sodass sich diese nicht weiter verschärfen. Eine (selbst)kritische Bestandsaufnahme und Reflexion der einzelnen Schulen bzw. eine Aufnahme dieser Themen in die schulische Qualitätsentwicklung wären hierfür wichtige Schritte.

Zweitens ist – auch darauf weisen die Ergebnisse der vorliegenden Studie in Übereinstimmung mit bestehenden Studien (Schmid et al. 2017; Ackeren et al. 2020) hin – zu berücksichtigen, dass die technische Ausstattung zwar eine notwendige, aber keine hinreichende Voraussetzung für erfolgreiches Distance Learning sein kann. Vielmehr bedarf es einer Reihe an digitalen Kompetenzen, die Schüler*innen dazu befähigen, mit digitalen Medien zu lernen. In diesem Zusammenhang erscheint wichtig, eine gewisse Verbindlichkeit zu erreichen – und zwar spätestens ab Beginn der Sekundarstufe –, um zu vermeiden, dass nur jenen Schüler*innen, die mit günstigen Ausgangsbedingungen ausgestattet sind, digitale Kompetenzen vermittelt werden.

Drittens ist darauf hinzuweisen, dass auch digitale Kompetenzen auf Seiten der Lehrer*innen eine wichtige Rolle spielen. Unterrichtsqualität und damit der Lernerfolg von Schüler*innen im digitalisierten Unterricht und Distance Learning sind nicht allein mit einer erfolgreichen Veränderung der ‚Oberflächenstruktur‘ sichergestellt – also schlicht der Umstellung auf digitale Tools. Vielmehr muss das Augenmerk darauf gelegt werden, kognitive Aktivierung der Schüler*innen, Unterstützung und Strukturierung auch bei der Nutzung digitaler Medien im Unterricht sicherzustellen (Voss und Wittwer 2020, S. 605ff.). Die Bedeutung dessen zeigen die hier vorgestellten Ergebnisse vor allem auch für jene Schüler*innen, die im Distance Learning kaum auf elterliche Unterstützungsleistungen zurückgreifen können.

## Footnotes

1 Für diese Veränderungen im Unterrichtsgeschehen werden im Folgenden die Begriffe ‚Distance Learning‘ und ‚Fernunterricht‘ synonym verwendet. Andere Begriffe wie ‚Homeschooling‘ werden in Anlehnung an die Ausführungen von Helm et al. (2021a, S. 239f.) vermieden, da diese u. a. mit bewusst gewähltem Heimunterricht assoziiert sind.

2 Für einen Überblick zu den unterschiedlichen Modellen und Ansätzen der Erklärung digitaler Ungleichheit siehe Zillien (2009, S. 90–125).

3 Allgemein gilt es bei der technischen Ausstattung zwischen den verfügbaren digitalen Medien, der Software und der Verfügbarkeit einer Internetverbindung im häuslichen Umfeld und im schulischen Kontext zu unterscheiden. Der Fokus der Studie, die diesem Beitrag zugrunde liegt, lag auf der technischen Ausstattung der Schüler*innen, die ihnen für die Phasen des Fernunterrichts zuhause zur Verfügung stand. Zu einer kritischen Auseinandersetzung mit der technischen Ressourcenausstattung österreichischer Schulen siehe beispielsweise Lassnigg (2021) sowie allgemein Huber et al. (2020, S. 47f.).

4 Davon wurden 15 Interviews mit Lehrer*innen bzw. Schulleiter*innen der Primarstufe geführt; die restlichen mit Lehrer*innen weiterführender Schulen: Drei Interviews mit Lehrer*innen aus (Neuen) Mittelschulen (NMS/MS) und vier mit Lehrer*innen aus Gymnasien (AHS); eines der Interviews wurde mit einer Lehrerin einer berufsbildenden höheren Schule (BHS) geführt, um diese Perspektive ergänzend einzubringen.

5 Ein Schwerpunkt der Erhebung lag auf dem Bereich Schulsozialarbeit; daneben wurden weiterer Akteur*innen aus dem breiten Feld schulischer Unterstützungssysteme interviewt: Schulpsycholog*innen, Schulärzt*innen, Schulassistent*innen, Jugendcoaches, Beratungslehrer*innen, Mitarbeiter*innen externer Lernhilfen, Freizeitpädagog*innen und Akteur*innen aus dem Bereich der flexiblen Hilfen.

6 Aufgrund der während der Projektlaufzeit weiterhin bestehenden Covid-Maßnahmen war eine Rekrutierung der Schüler*innen über die Schulen selbst nur in einzelnen Fällen möglich; daher wurden Gruppeninterviews u. a. in verschiedenen Jugendzentren (JuZ) geführt, um auch Kinder und Jugendliche aus strukturell benachteiligten Familien zu erreichen.

7 Die Fertigkeit, Wissen und Kenntnis zu vermitteln, ist ein eigener Problempunkt, der in den Interviews erwähnt wird – und der zunächst einmal alle Schüler*innen unabhängig vom sozialen Hintergrund betrifft: „Weil die Lehrerin halt viel besser erklären kann als die Mama. Dann versteht man das halt nicht so“, drückt es eine Schülerin im Interview aus (KiJu_02_VS, 17), und auch Lehrer*innen berichteten davon, dass den Eltern gewisse didaktische Fähigkeiten fehlten.
